# Genetic diversity and breed identification of Chinese and Vietnamese local chicken breeds based on microsatellite analysis

**DOI:** 10.1093/jas/skad182

**Published:** 2023-06-04

**Authors:** Zhong Zhuang, Long Zhao, Weicheng Zong, Qixin Guo, Xiaofan Li, Yulin Bi, Zixiu Wang, Yong Jiang, Guohong Chen, Bichun Li, Guobin Chang, Hao Bai

**Affiliations:** Key Laboratory for Animal Genetics & Molecular Breeding of Jiangsu Province, College of Animal Science and Technology, Yangzhou University, Yangzhou 225009, China; Key Laboratory for Animal Genetics & Molecular Breeding of Jiangsu Province, College of Animal Science and Technology, Yangzhou University, Yangzhou 225009, China; College of Innovation and Entrepreneurship, Yangzhou University, Yangzhou 225009, China; Key Laboratory for Animal Genetics & Molecular Breeding of Jiangsu Province, College of Animal Science and Technology, Yangzhou University, Yangzhou 225009, China; Key Laboratory for Animal Genetics & Molecular Breeding of Jiangsu Province, College of Animal Science and Technology, Yangzhou University, Yangzhou 225009, China; Joint International Research Laboratory of Agriculture and Agri-Product Safety, the Ministry of Education of China, Institutes of Agricultural Science and Technology Development, Yangzhou University, Yangzhou 225009, China; Key Laboratory for Animal Genetics & Molecular Breeding of Jiangsu Province, College of Animal Science and Technology, Yangzhou University, Yangzhou 225009, China; Key Laboratory for Animal Genetics & Molecular Breeding of Jiangsu Province, College of Animal Science and Technology, Yangzhou University, Yangzhou 225009, China; Key Laboratory for Animal Genetics & Molecular Breeding of Jiangsu Province, College of Animal Science and Technology, Yangzhou University, Yangzhou 225009, China; Key Laboratory for Animal Genetics & Molecular Breeding of Jiangsu Province, College of Animal Science and Technology, Yangzhou University, Yangzhou 225009, China; Joint International Research Laboratory of Agriculture and Agri-Product Safety, the Ministry of Education of China, Institutes of Agricultural Science and Technology Development, Yangzhou University, Yangzhou 225009, China; Key Laboratory for Animal Genetics & Molecular Breeding of Jiangsu Province, College of Animal Science and Technology, Yangzhou University, Yangzhou 225009, China; Joint International Research Laboratory of Agriculture and Agri-Product Safety, the Ministry of Education of China, Institutes of Agricultural Science and Technology Development, Yangzhou University, Yangzhou 225009, China; Key Laboratory for Animal Genetics & Molecular Breeding of Jiangsu Province, College of Animal Science and Technology, Yangzhou University, Yangzhou 225009, China; Joint International Research Laboratory of Agriculture and Agri-Product Safety, the Ministry of Education of China, Institutes of Agricultural Science and Technology Development, Yangzhou University, Yangzhou 225009, China; Key Laboratory for Animal Genetics & Molecular Breeding of Jiangsu Province, College of Animal Science and Technology, Yangzhou University, Yangzhou 225009, China; Joint International Research Laboratory of Agriculture and Agri-Product Safety, the Ministry of Education of China, Institutes of Agricultural Science and Technology Development, Yangzhou University, Yangzhou 225009, China

**Keywords:** breed identification, chicken, genetic diversity, microsatellite

## Abstract

South Asia and Southeast Asia are the origins of domestic chickens and are rich in poultry genetic resources, resulting in many unique local chicken breeds. However, with the rapid intensification of poultry farming worldwide, many local chicken breeds are threatened with extinction. In response to China’s “One Belt, One Road” policy, it is imperative to strengthen the conservation and breeding of local chicken breeds in China and Vietnam. This study characterized 18 microsatellite molecular genetic markers to analyze the genetic diversity of 21 local chicken populations in southern China (Yunnan and Guangxi Provinces) and Vietnam, breed identification tags for microsatellite loci were constructed. The results showed that a total of 377 alleles were detected in all breeds, and the most alleles (44) and the highest polymorphic information content (0.7820) were detected at the LEI0094 locus. The average polymorphic information content (**PIC**) content of the whole population was 0.65, indicating moderate polymorphism. The genetic diversity of the whole population was rich, except for two loci MCW0111 and MCW0016, that showed heterozygote excess at microsatellite loci, and the population had high genetic differentiation. The Vietnamese breeds showed low pairwise fixation coefficient (**FST**) and Nei’s standard genetic distance (**DS**) between them. According to the neighbor-joining dendrogram constructed by DS and the analysis of population genetic structure using the structure program, Longshengfeng chicken, Yunlong dwarf chicken, Tengchong white chicken, Xiayan chicken, and Daweishan mini chicken are similar, and Xishuangbanna game fowl, Wuding chicken, and Lanping silky chicken are similar to Yanjin black-bone chicken. In addition, excluding Dongtao chicken, other Vietnamese breeds are clustered together, indicating that the southern chicken breeds are closely related and have experienced better breeding. Overall, the whole population is rich in genetic resources, and the chicken breeds in the three regions are genetically close because of geographical factors and human activities. Dongtao chicken in Vietnamese, Chinese Yunnan local chicken breeds (*Gallus gallus* spadiceus), and red jungle fowl chickens (*Gallus gallus*) may have the same origin. We also constructed unique microsatellite molecular markers for 20 cultivars using 15 microsatellite loci. This study provides valuable insights to facilitate breed identification, improve cultivar protection, and new germplasm construction.

## Teaser

We analyzed the genetic diversity of local chicken breeds in southern China and Vietnam using microsatellite technology and described the affinities of these chicken breeds. Moreover, unique molecular tags were constructed for these breeds through unique allelic loci.

## Introduction

As an important component of animal genetic diversity, poultry plays a key role in the survival and development of humans. First, the meat and eggs provided overcome the most basic problem of food resources, and their genetic diversity also plays an important role in the ecological environment, material circulation, and soil water sources (Liao et al., 2016). Chickens are the most abundant poultry and the most consumed animals worldwide (Liu et al., 2006), as shown by the strong growth of poultry production globally (Aboe et al., 2006). Chickens are not only an important source of protein to meet human needs but also a critical economic source for low-income farmers in Asia, Africa, and Latin America (Missohou et al., 2002).

Both southern China and Vietnam are important places for domestication of the red jungle fowl (Storey et al., 2019), and a large number of sculptures of early stone-age chickens have been found by archeologists, demonstrating the ancient existence and importance of domesticated chickens (Berthouly et al., 2009; Huo et al., 2014). Yunnan and Guangxi Provinces in China and Vietnam are adjacent to each other and the region has complex terrain with many mountains, plateaus, and basins, pleasant climate, abundant rainwater, fertile land, and a rich biodiversity (Qu et al., 2006; Liao et al., 2016), Long-term material exchange between the three regions has created richer genetic resources (Granevitze et al., 2007; Berthouly et al., 2009; Schou et al., 2010).

Living in different ecological and feeding conditions, pheasants have been bred through long-term natural selection or non-systematic artificial selection to form unique local chicken breeds with different shapes and stable inheritance. Due to its long history of breeding or cultivation, it has better adaptability to local conditions. For example, *Gallus gallus* spadiceus, Daweishan mini chickens, Lanping silky chicken, Tengchong white chicken, and Yao chicken from Yunnan and Guanxi, China, and Dongtao chicken in Vietnam, have a unique appearance and also a special medicinal and ornamental value (Higham et al., 2011; Huo et al., 2014). Over the last few decades, some commercial varieties and their hybrids have met local economic needs in a relatively short period of time (Miao et al., 2013; Nguyen Van et al., 2020), followed by a gradual decline in the genetic diversity of chicken breeds. In addition, since the “Belt and Road Initiative” was proposed in 2013, China has strengthened its cooperation and exchange with neighboring countries (Tang et al., 2017). To promote the exchange of germplasm resources and the creation of new germplasms in China and Vietnam, it is particularly important to study the genetic diversity and gene exchange of chicken breeds between the two countries (Ullah et al., 2021). In this study, the genetic diversity of particular chicken breeds in southern China and Vietnam was analyzed to protect the genetic resources of local chicken breeds and establish the construction basis for improved exchange and breeding of better poultry breeds in the two countries.

Microsatellites are an effective diversity analysis tool, they are widely distributed in the genomes of eukaryotes (Brace et al., 1994; Pierszalowski et al., 2013) and have a high degree of conservation and generality, easy identification, wide distribution, high polymorphism, good reproducibility, can distinguish heterozygotes, and are very easy to mark and detect genetic material and traits between individuals and populations (Liu et al., 2006; Muchadeyi et al., 2007; Wilkinson et al., 2012). Many studies have shown that they play an important role in analyzing the genetic diversity of poultry, and studying their origin, evolutionary direction, and existing crises and causes (Koshiishi et al., 2020; Soglia et al., 2021; Zhang et al., 2021). At present, there are few reports on the genetic diversity of chicken breeds with characteristics in southern China (Yunnan, Guangxi) and Vietnam, and the genetic resources in this area are quite rich; therefore, it is necessary to explore the diversity of chicken breeds. In addition, microsatellites have played an increasingly important role in the wildlife conservation (Moura et al., 2017), paternity testing (Correia et al., 2021), and meat source traceability (Kannur et al., 2017) in recent years. Due to the close geographical location, some chicken breeds are difficult to distinguish by shape and are rare breeds, therefore to protect the needs of breeds, molecular tags are needed for more accurate breed identification (de Melo et al., 2020). This study not only helps to understand the origin of domestic chickens and genetic differences of these breeds in this region but also assists in the evaluation of breeding potential and provides a basis for realizing the construction of new germplasm under the premise of meeting the protection of germplasm resources.

## Material and Methods

### Ethical approval

All the experimental procedures were conducted in strict accordance with the guidelines approved by the China Council on Animal Care and the Ministry of Science and Technology of the People’s Republic of China. In addition, all experimental birds were managed and handled according to the guidelines approved by the Animal Care and Use Committee of Yangzhou University (No.: YZUDWSY2017-11-07).

### Sampling and microsatellite loci

**Figure 1.**
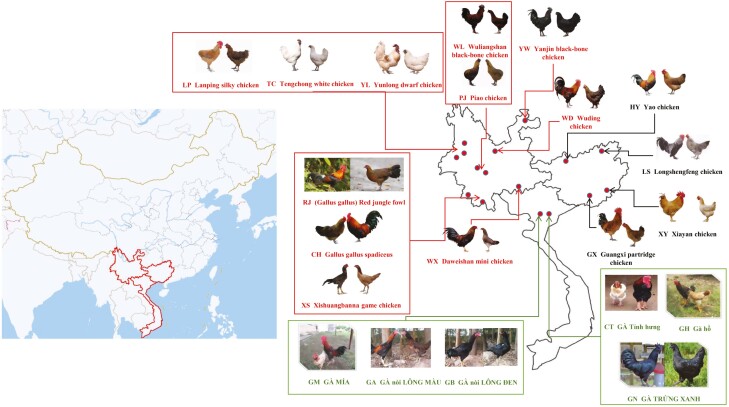
Geographical distribution of 21 breeds of local chickens used in this study in China and Vietnam. Red auxiliary line and font represent local chicken breeds in Yunnan, black auxiliary line and font represent local chicken breeds in Guangxi, China, and green auxiliary line and font represent local chicken breeds in Vietnam.

The present study included 534 birds from 21 different populations; 354 belonged to 15 Chinese chicken breeds (including 30 birds of red jungle fowl). In total, 180 birds were sourced from six Vietnamese local chickens. The geographical location of the chicken populations is shown in Table 1 and Figure 1. Among these, most of the Chinese chicken breeds are endangered, especially Piao, Tengchong white, Daweishan mini, Yunlong dwarf, and Yanjin black-bone chickens, whose genetic resources are relatively scarce. At present, most are bred in local breeding farms established by the state, and the samples collected in this study were provided by these farms. The six chicken breeds in Vietnam were raised via large-scale farming on DABACO Company breeding farms. Samples were reared separately in corresponding populations without interaction with each other.

**Table 1.** Basic information on chicken genetic resources used in this study

Breed^1^	Abbreviation	Economic character	Habitat	Sample size
^a^Red jungle fowl (Gallus gallus)	RJ	——	Menghai County, Yunnan Province	30
^a^ *Gallus gallus* spadiceus	CH	Meat type	Xishuangbanna Dai Autonomous Prefecture, Yunnan Province	30
^b^Yao chicken	HY	Meat type	Nandan county, Guangxi	28
^b^Guangxi partridge chicken	GX	Meat and Egg type	Lingshan County, Guangxi	26
^b^Longshengfeng chicken	LS	Meat type	Longsheng County, Guangxi	26
^a^Piao chicken	PJ	Meat and Egg type	Zhenyuan County, Yunnan Province	12
^a^Tengchong white chicken	TC	Meat and Medicinal type	Tengchong County, Yunnan Province	18
^a^Daweishan mini chicken	WX	Meat and Ornamental type	Pingbian County, Yunnan Province	15
^a^Wuding chicken	WD	Meat type	Wuding County, Yunnan Province	28
^a^Xishuangbanna game chicken	XS	Meat and Ornamental type	Xishuangbanna Dai Autonomous Prefecture, Yunnan Province	25
^a^Yunlong dwarf chicken	YL	Meat and Egg type	Yunlong County, Yunnan Province	19
^a^Lanping silky chicken	LP	Meat and Medicinal type	Lanping County, Yunnan Province	28
^b^Xiayan chicken	XY	Meat type	Rong County, Guangxi	24
^a^Wuliangshan black-bone chicken	WL	Meat and Egg type	Jingdong Yi Autonomous County, Pu’er City, Yunnan Province	30
^a^Yanjin black-bone chicken	YJ	Meat and Egg type	Yanjin County, Yunnan Province	15
^c^GÀ Tỉnh hưng	CT	Meat type	Tỉnh hưng yên (Khingan Province, Vietnam)	30
^c^GÀ MÍA	GM	Meat type	Vietnam	30
^c^GÀ nòi LÔNG MÀU	GA	Meat and Egg type	Vietnam	30
^c^GÀ nòi LÔNG ĐEN	GB	Meat type	Vietnam	30
^c^Gà hồ	GH	Meat and Egg type	Vietnam	30
^c^GÀ TRÚNG XANH	GN	Meat type	Vietnam	30
Total				534

^1^One asterisk (^a^) indicates the Yunnan chicken breed in China, two asterisks (^b^) indicate the Guangxi chicken breed in China, and three asterisks (^c^) indicate the Vietnamese chicken breed.

A total of 18 microsatellite markers (MCW0081, MCW0248, MCW0213, ADL0278, MCW0067, MCW0330, MCW0165, MCW0020, MCW0103, ADL0268, MCW0016, MCW0098, MCW0123, LEI0094, LEI0166, MCW0111, MCW0104, and MCW0183) were analyzed to estimate various parameters of genetic diversity. These loci are recommended by the Food and Agriculture Organization of the United Nations (FAO) (Organization, 2012) for the analysis of genetic diversity in chicken breeds.

### DNA extraction and PCR-based profiling

Blood samples of Chinese local chicken breeds were provided by the Poultry Institute, Chinese Academy of Agricultural Sciences, and Jiangsu Institute of Poultry Sciences. The total DNA used for polymerase chain reaction (PCR) amplification was extracted from the blood specimens. The mixture was obtained using 800 μL lysis buffer (20 mM Tris, 400 mM NaCl, 35 mM SDS, 6 mM EDTA, PH 8.0) and was added to 50 μL of EDTA whole blood sample. The whole tube was centrifuged, and the pellet was suspended in 30 μL Proteinase K (20 mg/mL). After overnight incubation at 55 °C, the proteins were removed using Tris-saturated phenol and chloroform, and the DNA was precipitated with ethanol. DNA samples from five local Vietnamese chicken breeds were obtained from DABACO, Vietnam. The quality and quantity of the DNA extracts were detected using a nucleic acid ­analyzer ­(NANODROP ND-1000, Thermo Fisher Scientific, MA, USA), and each DNA extract was diluted to 50 ng/µL.

Detailed information on the 18 microsatellite primer pairs is shown in Table 2. All primers were synthesized by the Shanghai Bioengineering Company (Fedbio, Shanghai, China) and labeled with FAM (absorption wavelength 494 nm, emission wavelength 522 nm, blue), HEX (absorption wavelength 535 nm, emission wavelength 556 nm, green), and ROX (6-carboxy-x-rhodamine, absorption wavelength 587 nm, emission wavelength 607 nm, red) fluorescent dye at the 5ʹ ends. PCR amplification was performed with minor modifications based on the amplification conditions of the original primers.

**Table 2.** Details of considered microsatellite loci

Locus	Primer sequence (5ʹ-3ʹ)	Fluorescent label^1^	Size range (bp)	Annealing temp (C)
MCW0081	GTTGCTGAGAGCCTGGTGCAG CCTGTATGTGGAATTACTTCTC	FAM	108~143	55
MCW0248	GTTGTTCAAAAGAAGATGCATG TTGCATTAACTGGGCACTTTC	HEX	212~225	55
MCW0213	CTGTTCACTTTAAGGACATGG GACAAGTCAACAACTTGCCAG	Rox	266~318	60
ADL0278	CCAGCAGTCTACCTTCCTAT TGTCATCCAAGAACAGTGTG	FAM	105~126	56
MCW0067	GCACTACTGTGTGCTGCAGTTT GAGATGTAGTTGCCACATTCCGAC	HEX	173~182	56
MCW0330	TGGACCTCATCAGTCTGACAG AATGTTCTCATAGAGTTCCTGC	Rox	250~288	56
MCW0165	CAGACATGCATGCCCAGATGA GATCCAGTCCTGCAGGCTGC	FAM	92~116	60
MCW0020	TCTTCTTTGACATGAATTGGCA GCAAGGAAGATTTTGTACAAAATC	HEX	177~188	56
MCW0103	AACTGCGTTGAGAGTGAATGC TTTCCTAACTGGATGCTTCTG	Rox	267~273	55
ADL0268	CTCCACCCCTCTCAGAACTA CAACTTCCCATCTACCTACT	FAM	100~120	56
MCW0016	ATGGCGCAGAAGGCAAAGCGATAT TGGCTTCTGAAGCAGTTGCTATGG	HEX	127~157	56
MCW0098	GGCTGCTTTGTGCTCTTCTCG CGATGGTCGTAATTCTCACGT	Rox	241~263	60
MCW0123	CCACTAGAAAAGAACATCCTC GGCTGATGTAAGAAGGGATGA	FAM	72~94	56
LEI0094	GATCTCACCAGTATGAGCTGC TCTCACACTGTAACACAGTGC	HEX	241~287	58
LEI0166	CTCCTGCCCTTAGCTACGCA TATCCCCTGGCTGGGAGTTT	Rox	334~367	58
MCW0111	GCTCCATGTGAAGTGGTTTA ATGTCCACTTGTCAATGATG	FAM	91~112	55
MCW0104	TAGCACAACTCAAGCTGTGAG AGACTTGCACAGCTGTGTACC	HEX	188~236	56
MCW0183	ATCCCAGTGTCGAGTATCCGA TGAGATTTACTGGAGCCTGCC	Rox	284~332	60

^1^FAM, HEX, and ROX present blue, green, and red, respectively, during genotyping with fluorescent markers.

The PCR amplifications were carried out in 20 μL total volumes, containing 2 μL of primers (10 μm each forward and reverse), 10 μL of 2 × Taq Master Mix (Dye Plus), 1 μL of DNA template, and 7 μL of triple-distilled water. The PCR conditions consisted of an initial denaturation at 95 °C for 5 min, followed by 35 cycles of denaturation at 95 °C for 30 s, annealing temperature varied between 55 and 60 °C for 45 s, and extension at 72 °C for 40 s, with a final extension step at 72 °C for 10 min, last saved at 4 °C. The amplified products were analyzed using 1% agarose gel electrophoresis. The qualified amplicons were sent to the Shanghai Bioengineering Company (Fedbio, Shanghai, China) and sequenced using an analyzer (ABI3730, Thermo Fisher Scientific, CA, USA). Microsatellite genotyping criteria were as follows: when the gene was ­homozygous, it was unimodal in the genotype map; when the gene was interested in heterozygotes, the main peak in the genotyping map was bimodal.

### Statistical analysis

Values such as peak size, fragment length, peak area, and homozygous (unimodal) or heterozygous (bimodal) states were analyzed using GeneMapper4.0 software. The observed number of alleles (Na), effective number of alleles (Ne), observed heterozygosity (Ho), expected heterozygosity (He), and polymorphism information content (PIC) of each marker were estimated using the Excel Microsatellite-Toolkit (https://www.researchgate.net/profile/Benjamin-Barth-2/post/Where-can-I-download-the-excel-micro-satellite-toolkit/attachment/59d635dac49f478072ea3998/AS%3A273668205154304%401442258992481/download/MStools.zip).

VPOPGENE 1.32 was used to generate Wright F-statistics (FIS, FIT, and FST) and Nei’s standard genetic distance (DS). Neighbor-joining dendrograms were constructed using the unweighted group average unweighted pair-group method with arithmetic mean (UPGMA) based on DS using the MEGA7 software (Nei, 1972). Principal coordinate analysis (PCoA) analysis was performed with GenAlex 6.5 (Peakall and Smouse, 2012). The Structure 2.3.4 software was used to construct a genetic structure diagram of 21 populations according to the number of genomes and to divide the entire population into K small groups. For each K value, we repeated the Structure program 10,000 times, each time with 100,000 burn-in operations to construct the population genetic structure map.

## Results

### Microsatellite polymorphism and population genetic diversity

Results from molecular genetic characterization of 21 local chicken breeds using 534 samples and 18 microsatellites showed that are provided in Table 3. Eighteen microsatellite loci detected a total of 377 alleles in 21 populations, with an average of 20.944 alleles per locus, and the number of alleles per locus ranged from 6 (MCW0103) to 44 (LEI0094); all loci were polymorphic. LEI0094 had the highest polymorphic information content of 0.782, and mean PIC of 0.647, indicating high polymorphism. The average Ho at 18 loci was 0.609, which was lower than the average He of 0.699. The average within-subpopulation inbreeding coefficient (FIS) was 0.1080, except for two loci (MCW0016 and MCW0111) that yielded negative FIS values; other loci had some degree of inbreeding in the subpopulation. In addition, the average total population inbreeding coefficient (FIT) was 0.2569 and the average gene fixation coefficient (FST) was 0.1670, indicating that genetic differentiation between populations was large.

**Table 3.** Genetic polymorphism statistics at eighteen microsatellite loci in twenty-one chicken breeds.

Locus	Na	Ne	Ho	He	PIC	FIS	FIT	FST	Nm^1^
MCW0081	21	2.1978	0.515	0.545	0.488	0.0356	0.1753	0.1448	1.4766
MCW0248	14	3.1646	0.584	0.684	0.632	0.1278	0.2607	0.1524	1.3903
MCW0213	33	5.1813	0.685	0.807	0.769	0.1327	0.2559	0.1420	1.5107
ADL0278	16	2.9155	0.562	0.657	0.607	0.1244	0.3212	0.2248	0.8621
MCW0067	10	3.7594	0.620	0.734	0.673	0.1366	0.2761	0.1617	1.2965
MCW0330	19	4.065	0.737	0.754	0.701	0.0010	0.1482	0.1473	1.4475
MCW0165	11	2.2523	0.280	0.556	0.487	0.4850	0.6387	0.2985	0.5876
MCW0020	12	3.2362	0.635	0.691	0.637	0.0578	0.2596	0.2142	0.9174
MCW0103	6	2.5575	0.468	0.609	0.532	0.2091	0.3937	0.2334	0.8209
ADL0268	16	3.9683	0.598	0.748	0.694	0.1812	0.2913	0.1345	1.6094
MCW0016	28	5.5556	0.819	0.820	0.777	-0.0208	0.0971	0.1155	1.9151
MCW0098	10	1.5361	0.264	0.349	0.298	0.2237	0.5438	0.4124	0.3562
MCW0123	23	4.717	0.768	0.788	0.743	0.0030	0.1273	0.1246	1.7562
LEI0094	44	5.5249	0.753	0.819	0.782	0.0592	0.1865	0.1353	1.5978
LEI0166	20	3.3113	0.594	0.698	0.643	0.1258	0.2523	0.1446	1.4785
MCW0111	22	4.9751	0.791	0.799	0.753	−0.0122	0.0954	0.1063	2.1017
MCW0104	34	4.2017	0.740	0.762	0.714	0.0032	0.1246	0.1218	1.8031
MCW0183	38	4.1152	0.543	0.757	0.718	0.2673	0.3428	0.1031	2.1758
Average	20.944	3.7350	0.609	0.699	0.647	0.1080	0.2569	0.1670	1.2472

Abbreviations: ^1^Nm, gene flow estimated from FST = 0.25(1−FST)/FST.

### Comparison of genetic diversity among different breeds

For the investigated chicken breeds, the lowest Ne value (5.22) was detected in WX and the highest value (9.22) was detected in XY. Ho ranged from 0.4623 (LP) to 0.7193 (YL), He ranged from 0.5964 (CH) to 0.7920 (XY), and PIC ranged from 0.5531 (CH) to 0.7425 (XY) for all the breeds. Six Vietnamese landrace breeds (7.66) had slightly larger mean Ne values than the Chinese landrace breeds (6.27), but the mean heterozygosity (Vietnamese landrace breeds 0.6794, Chinese landrace breeds 0.6101) and PIC (Vietnamese landrace breeds 0.6364, Chinese landrace breeds 0.6214) were not significantly different. In addition to CT, the parameters of the five Vietnamese local chicken breeds were higher than those of the Chinese local chicken breeds and were maintained at a higher level (Ho = 0.6063 He = 0.6700 PIC = 0.6484) (Table 4).

**Table 4.** Genetic polymorphism parameters according to studied chicken breeds across eighteen microsatellite loci

Breed	Ne (mean ± SD)	Ho (mean ± SD)	He (mean ± SD)	PIC (mean)
CT	6.22 ± 2.34	0.5315 ± 0.0215	0.6292 ± 0.0403	0.5764
CH	6.11 ± 2.08	0.4907 ± 0.0215	0.5964 ± 0.0466	0.5531
WL	7.11 ± 2.76	0.5361 ± 0.0215	0.6806 ± 0.0357	0.6292
LS	8 ± 4.01	0.6726 ± 0.0218	0.7121 ± 0.0475	0.6648
YL	8.22 ± 3.51	0.7193 ± 0.0243	0.7662 ± 0.0372	0.7159
TC	7.28 ± 3.32	0.7035 ± 0.0254	0.7339 ± 0.0201	0.6800
XY	9.22 ± 3.67	0.6388 ± 0.0232	0.7920 ± 0.0201	0.7425
WX	5.22 ± 2.51	0.5148 ± 0.0306	0.6250 ± 0.0628	0.5625
RJ	7 ± 2.61	0.6607 ± 0.0204	0.7163 ± 0.0276	0.6611
HY	7.61 ± 2.87	0.6329 ± 0.0215	0.7362 ± 0.0224	0.6853
GX	8.83 ± 3.82	0.6453 ± 0.0221	0.7531 ± 0.0275	0.7069
PJ	5.67 ± 2.14	0.6549 ± 0.0324	0.7209 ± 0.0288	0.6453
XS	7.33 ± 3.27	0.6089 ± 0.023	0.7152 ± 0.0276	0.6612
WD	6.5 ± 2.85	0.6151 ± 0.0217	0.6599 ± 0.0381	0.6027
LP	6.44 ± 2.64	0.4623 ± 0.0222	0.6565 ± 0.0291	0.6015
YJ	6.56 ± 2.53	0.6634 ± 0.029	0.7231 ± 0.0313	0.6592
GA	8.56 ± 3.81	0.6413 ± 0.0207	0.7263 ± 0.0443	0.6876
GB	8.17 ± 3.81	0.6426 ± 0.0206	0.6700 ± 0.0407	0.6266
GM	7.83 ± 3.71	0.6278 ± 0.0208	0.6966 ± 0.0489	0.6589
GN	8.33 ± 3.48	0.5955 ± 0.0211	0.7065 ± 0.0405	0.6648
GH	6.83 ± 2.73	0.525 ± 0.0215	0.6476 ± 0.0553	0.6042

### Standard genetic distance and neighbor-joining dendrogram among flocks

FST and the DS between populations can reflect the degree of variation and differentiation. FST and genetic distances between the different populations are shown in Table 5. The FST in the investigated population was significant, between 0.0110 and 0.1834, with the highest FST found between WX and GH, and the lowest degree of genetic differentiation between CT in Vietnam and GX in China, while other Vietnamese breeds GA, GB, and GH had the smallest genetic variation with GM and GN, respectively. Genetic distances between breeds ranged between 0.0519 and 1.3638. The genetic distance was consistent with FST, the largest genetic distance emerged between the Vietnamese breeds GH and WX (1.3638), and the smallest genetic distance among all breeds was between the Vietnamese local chicken breeds GA and GM (0.0519). Overall, the closest neighbors of the Vietnamese breed CT in the Chinese breed were GX, CH, and WL (FST: 0.0721, 0.0743, 0.0762), and genetic relationships were similar between the other five Vietnamese breeds, relatively distant from Chinese local chicken breeds.

**Table 5.** Estimated mean FST (below diagonal) and DS (above diagonal) for each pair of populations

Population	CT	CH	WL	LS	YL	TC	XY	WX	RJ	HY	GX	PJ	XS	WD	LP	YJ	GA	GB	GM	GN	GH
CT	–	0.287	0.34	0.6648	0.691	0.7482	0.6711	0.8916	0.561	0.4687	0.3805	0.5105	0.7982	0.7858	0.8839	0.8505	0.5199	0.5771	0.489	0.642	0.68
CH	0.0762	-	0.2947	0.6619	0.7564	0.8159	0.6079	1.0872	0.5775	0.4549	0.2423	0.5335	0.7185	0.7105	0.8613	0.7556	0.4412	0.4495	0.3501	0.4926	0.5096
WL	0.0743	0.0715	-	0.7087	0.7501	0.7278	0.528	1.0529	0.6596	0.4681	0.3794	0.5453	0.8082	0.7458	0.7624	0.7517	0.3714	0.4054	0.3342	0.5125	0.5693
LS	0.1124	0.1196	0.1052	–	0.1322	0.1987	0.2269	0.2621	0.7192	0.686	0.703	0.6691	0.8993	1.0151	0.9372	0.7655	0.8199	0.9968	0.8952	0.9823	1.1363
YL	0.1053	0.1181	0.0986	0.0237	–	0.1696	0.2106	0.2265	0.7387	0.7272	0.7622	0.7065	0.9088	1.0156	0.9465	0.7024	0.7854	0.8548	0.8528	0.8259	1.0338
TC	0.1145	0.1297	0.1012	0.0356	0.0279	–	0.2054	0.3817	0.8078	0.7954	0.8453	0.83	1.0757	1.1207	1.0294	0.8643	0.9078	0.9427	1.0084	0.8815	1.1283
XY	0.0989	0.1005	0.0747	0.0363	0.0294	0.0314	–	0.4277	0.7588	0.5756	0.5449	0.6965	0.8659	0.927	0.9125	0.6848	0.6361	0.6979	0.6643	0.6713	0.8513
WX	0.1600	0.1829	0.1580	0.0605	0.0511	0.0779	0.0796	–	0.8898	1.0029	1.1284	0.852	1.1389	1.2717	1.1819	0.9287	0.9744	0.9763	1.0497	1.0084	1.3638
RJ	0.0999	0.1093	0.0994	0.0985	0.0904	0.1006	0.0862	0.1380	–	0.4246	0.4397	0.4677	0.7116	0.6482	0.7392	0.7208	0.5776	0.585	0.5362	0.6174	0.6604
HY	0.0857	0.0905	0.0759	0.0918	0.0858	0.0958	0.0689	0.1421	0.0649	–	0.1741	0.3476	0.5382	0.5481	0.5876	0.5689	0.3707	0.4561	0.3855	0.5206	0.5928
GX	0.0721	0.0565	0.0631	0.0902	0.0851	0.0958	0.0636	0.1426	0.0645	0.0286	–	0.336	0.6102	0.6501	0.7477	0.6508	0.3626	0.368	0.3342	0.4731	0.4693
PJ	0.0946	0.1022	0.0884	0.0964	0.0899	0.1053	0.0849	0.1346	0.0746	0.0577	0.0545	–	0.5477	0.5573	0.5348	0.5513	0.5168	0.5412	0.497	0.7561	0.8072
XS	0.1247	0.1250	0.1130	0.1129	0.1026	0.1184	0.0940	0.1557	0.0973	0.0777	0.0818	0.0844	–	0.1076	0.1448	0.1513	0.6123	0.7283	0.6446	0.6451	0.614
WD	0.1361	0.1364	0.1199	0.1340	0.1213	0.1337	0.1105	0.1772	0.1030	0.0890	0.0966	0.0945	0.0247	–	0.1291	0.2021	0.4896	0.6005	0.5149	0.6119	0.572
LP	0.1461	0.1528	0.1223	0.1296	0.1186	0.1299	0.1107	0.1737	0.1123	0.0939	0.1060	0.0929	0.0325	0.0321	–	0.1594	0.5959	0.7132	0.6594	0.6945	0.7031
YJ	0.1290	0.1275	0.1085	0.1024	0.0885	0.1063	0.0823	0.1403	0.0984	0.0812	0.0860	0.0912	0.0293	0.0431	0.0355	–	0.6304	0.7192	0.673	0.5968	0.6019
GA	0.0935	0.0900	0.0651	0.1040	0.0902	0.1036	0.0748	0.1392	0.0826	0.0572	0.0543	0.0771	0.0866	0.0838	0.0965	0.0891	–	0.1364	0.0519	0.2781	0.3998
GB	0.1105	0.0996	0.0778	0.1299	0.1084	0.1200	0.0916	0.1546	0.0940	0.0763	0.0633	0.0891	0.1086	0.1068	0.1181	0.1075	0.0290	–	0.1131	0.2206	0.4197
GM	0.0942	0.0796	0.0635	0.1160	0.1010	0.1164	0.0829	0.1510	0.0837	0.0632	0.0549	0.0793	0.0951	0.0916	0.1086	0.0978	0.0110	0.0256	–	0.2805	0.3786
GN	0.1110	0.1001	0.0858	0.1199	0.0975	0.1064	0.0816	0.1474	0.0902	0.0771	0.0697	0.1036	0.0933	0.1011	0.1102	0.0882	0.0487	0.0455	0.0522	–	0.1458
GH	0.1277	0.1128	0.1036	0.1422	0.1235	0.1348	0.1073	0.1834	0.1067	0.0959	0.0792	0.1168	0.1015	0.1077	0.1236	0.0979	0.0744	0.0853	0.0752	0.0337	–

**Figure 2.**
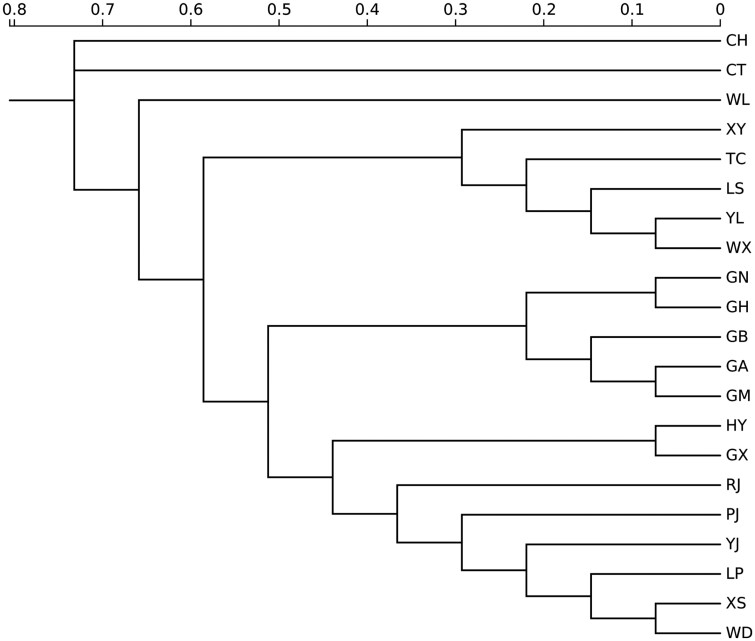
Genetic relationship among 21 local chicken populations. Phylogenetic tree of 21 chicken breeds by UPGMA based on DS.

Branches of the neighbor-joining dendrogram based on DS showed the genetic structure of Chinese and Vietnamese local chicken breeds. Branches of the neighbor-joining dendrogram based on DS show the genetic structure of Chinese and Vietnamese local chicken breeds. The dendrogram included four different clusters: XS and WD were first clustered and then clustered with LP, YJ, PJ, RJ, and finally clustered with HY and GX as the first cluster. The second cluster was formed by five Vietnamese cultivars; GA and GM were clustered and clustered with GB and finally clustered with GN and GH. YL and WX are aggregated with LS, TC, and XY in turn to form the third cluster. Finally, WL, CT, and CH which were far from other varieties in the genetic distance were clustered into the last cluster. We found that the Vietnamese chicken breed CT was closest to the Yunnan chicken breeds CH and WL in the genetic distance, while the genetic distance differences among other Vietnamese breeds were small and differences were small in genetic distance with the Guangxi chicken breeds HY, GX. The genetic distance between RJ and PJ in Yunnan, as well as HY and GX in Guangxi, was relatively close, which indicates that some chicken breeds in Yunnan, Guangxi, and Vietnam have certain gene exchanges and may have the same ancestors (Figure 2).

### Population structure analysis

**Figure 3.**
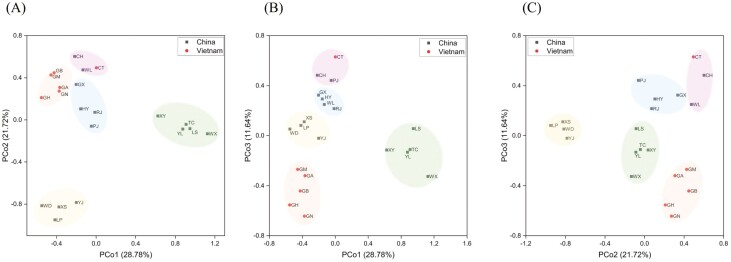
Principal coordinate analysis for 21 populations. (A) PCo1 and 2 together represent the majority of the genetic variation (~50.50%). (B) PCo1 and 3 together represent ~40.42% of the genetic variation. (C) PCo2 and 3 together represent ~33.36% of the genetic variation. Each dot represents a chicken breed and the circled chicken breeds are the most distinct chicken populations.

The relationship among 21 local chicken breeds was evaluated using principal coordinate analysis (PCoA). The results are shown in Figure 3. The plane distance scatter plot shows that all populations are divided into five subgroups. The first subgroup is CT from Vietnam and CH, PJ from Yunnan, China. The second subgroup is composed of GX, HY from Guangxi and GX, HY from Yunnan. The third subgroup consists of Chinese Yunnan varieties WD, LP, XS, and YJ. The fourth subgroup consisted of the Chinese Guangxi varieties XY, LS, and the Chinese Yunnan varieties TC, YL, and WX.

**Fig. 4.**
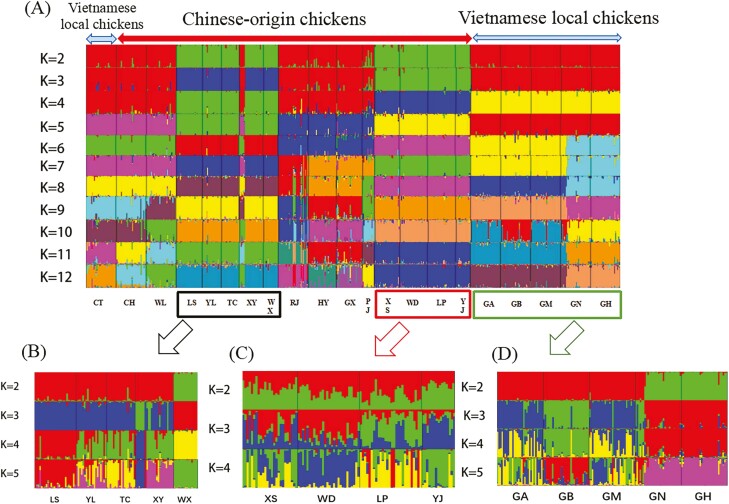
Structure analysis of 21 local chicken groups in Vietnam and China. (A) Cluster plots of 21 local chicken breeds in China and Vietnam, with three groups not separated, group 1 labeled with black auxiliary line, group 2 labeled with red auxiliary line, and group three labeled with green auxiliary line. (B) Cluster plot of subpopulations in group 1. (C) Cluster plot of subpopulations in group 2. (D) Cluster plot of subpopulations in group 3.

The population structure of 21 chicken groups was analyzed using Structure software. The results of the analysis are shown in Figure 4A. At *K* (the number of populations) = 2, six Vietnamese local chicken breeds formed the first group with RJ, CH, WL, HY, GX, and PJ, whereas other chicken breeds were divided into the second group. At *K* > 2, LS, YL, TC, XY, and WX were divided into one group, XS, WD, LP, and YJ were divided into one group, while Vietnamese local chickens and other Chinese chicken breeds were kept in the same group until the *K* value increased to four. When *k* = 5, the whole population was divided into five subpopulations, which were consistent with the PCoA segregation population. Under the condition *K* = 6, five Vietnamese landrace except CT were divided into two subgroups: breeds GA, GB, and GM were grouped into one group and breeds GN and GH were grouped into another. Until *K* = 12, the five Vietnamese chicken breeds (GA, GB, GM, GN, and GH) were not completely separated. At the same time, the second group was composed of LS, YL, TC, XY, and WX, and the third group which was composed of XS, WD, LP, and YJ, did not show separation. We performed secondary classification of these three subsets.

**Fig. 5.**
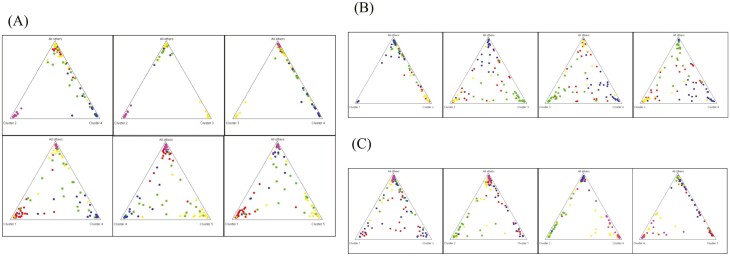
Distribution plots of independent populations of subgroups 1, 2, and 3. (A) Population distribution of five species in subgroup 1, Cluster1 (LS), Cluster2 (YL), Cluster3 (TC), Cluster4 (XY), Cluster5 (WX). (B) Population distribution of five species in subgroup 2, Cluster1 (XS), Cluster2 (WD), Cluster3 (LP), Cluster4 (YJ). (C) Population distribution of five species in subgroup 3, Cluster1 (GA), Cluster2 (GB), Cluster3 (GM), Cluster4 (GN), Cluster5 (GH).

As shown by the population genetic structure consisting of LS, YL, TC, XY, and WX (Figure 4B), WX first segregated at *K* = 2, LS at *K* = 4, and XY and TC at *K* = 5. At *K* = 5, the distribution plots of the independent groups of the five populations are shown in Figure 5A, where each individual in the YL population (Cluster 2) and TC population (Cluster 3) can be separated from the other populations, indicating that these two populations are relatively independent and distant from the other populations. However, the LS population (Cluster 1), XY population (Cluster 4), and WX population (Cluster 5) individuals were intermingled, and the population inheritance was close, of which the LS population (Cluster 1) was most closely related to the XY population (Cluster 4).

The second subgroup population genetic structure, consisting of XS, WD, LP, and YJ, is shown in Figure 4C, with no breed segregation at *K* = 2. YJ and LP were separated at *K* = 3, and WD at *K* = 4. The results of the independent group distribution of the four populations at *K* = 4 are shown in Figure 5B. All individuals of the XS population (Cluster 1) were distantly related to other populations, while the WD population (Cluster 2) was closely related to the YJ population (Cluster 3) as well as the LP population (Cluster 4), and there was confounding between individuals. Overall, the WD population (Cluster 2) was more distant from the YJ population (Cluster 3) than the LP population (Cluster 3) and YJ population (Cluster 4), the dispersion between each other was also relatively low.

Finally, the genetic structure map results for the five Vietnamese landraces are presented in Figure 4D, with the GA, GB, and GM breeds clustered together and the GN and GH breeds clustered together at *K* = 2. Breed GB was segregated from the population when *K* = 3, but until *K* = 5, breeds GN and GH remained as a whole. There were some boundary distinctions between the other groups. The independent group distribution of the five Vietnamese chicken populations at *K* = 5 showed that the Vietnamese chicken breed GB (Cluster 2), had less individual confounding with the other four populations, and individual confounding was more severe between populations GA (Cluster 1) and GM (Cluster 3) as well as between population GN (Cluster 4) and population GH (Cluster 5) (Figure 5C). The overall results showed that population GB was far from other populations, population GA was close to population GM, and population GM was close to population GN.

### Breed identification label development

To search for loci for specific breeds, we compared 21 local chicken breeds from Vietnam and China. Fifteen candidate breed-specific markers were identified, and unique alleles were detected at these loci in all breeds except Vietnamese GM (Table 6). MCW0183 had the most unique alleles among these breeds, with 11. CT, LS, WX, RJ, WD, LP, YJ, and GN had unique genotypes at this locus. MCW0165 had the fewest unique alleles and was present only in the RJ breeds. For all loci, 80 unique alleles were detected across 20 breeds, with unique allele types varying from 1 to 11 for each breed.

**Table 6.** Unique alleles^1^ in 21 chicken breeds

STR	CT	CH	WL	LS	YL	TC	XY	WX	RJ	HY	GX	PJ	XS	WD	LP	YJ	GA	GB	GN	GH
MCW0081			125/1.67		143/2.63				113/1.67	115/1.79	129/1.92								123/1.67	
			140/1.67						124/3.33										128/1.67	
MCW0213				295/1.92		316/2.78			266/13.33	317/1.79	288/1.92							313/1.67		
				297/1.92		318/2.78					305/5.77									
				312/3.85																
ADL0278							111/2.08					114/8.73			126/5.36					
MCW0330					278/2.63				280/1.67										250/1.67	
MCW0165									99/1.67											
MCW0020	177/3.33																			
ADL0268								104/3.33		116/1.79				120/1.79						
MCW0016					155/2.63		157/2.17		133/1.67				144/14.00					132/1.67		
MCW0098				263/3.85			253/2.08		241/1.67		255/1.92									
MCW0123																	72/1.67	92/6.67		
																	91/3.33	93/1.67		
LEI0094					275/2.63										285/1.79		241/1.67	247/3.33	243/1.67	
					286/2.63										287/1.79		270/1.67			
LEI0166					360/2.63			349/6.67	334/1.67											
									336/5.00											
									367/16.67											
MCW0111		107/1.67	93/1.67					91/3.33											108/8.33	
		112/3.33																		
MCW0104		200/3.33	194/1.67		234/2.63			215/26.67				236/4.17	231/2.00							197/5.17
			198/3.33																	
MCW0183	284/1.67			332/1.92				322/3.33	291/3.33					308/1.79	324/14.29	325/3.57			309/1.72	
	292/1.67																		323/1.72	
	293/3.33																			

^1^Two values are chosen to represent the characteristics of the breed’s unique alleles on this microsatellite; the left side of the symbol “/” in the table is the length of the unique allele at this microsatellite locus of the breed; the right side of the symbol “/” in the table is the probability of the unique allele of the breed.

As each chicken species has different unique allele types at different loci, the unique allele types of each breed can be used to construct an identification tag table for different chicken breeds. Each breed can be distinguished and identified on this basis, and the more unique allele tags are obtained by the breed, the more accurate the breed identification will be (Table 7). The results showed that the application of 15 markers in distinguishing varieties was successful and that the differentiation of all varieties was completed with the help of 15 markers, which laid a solid foundation for variety identification.

**Table 7.** Identified labels for different breeds^1^

Breeds	Labeling^2^	Breeds	Labeling
CT	⑥1⑮1⑮3⑮4	GX	①7②2②5⑨3
CH	⑬3⑬5⑭4	PJ	③2⑭8
WL	①5①8⑬2⑭1⑭3	XS	⑧3⑭6
LS	②3②4②6⑨4⑮11	WD	⑦3⑮5
YL	①9④2⑧4⑪5⑪7⑫4⑭7	LP	③3 ⑪6⑪8⑮9
TC	②8②10	YJ	⑮10
XY	③1⑧5⑨2	GA	⑩1⑩2⑪1⑪4
WX	⑦1⑫3⑬1⑭5⑮7	GB	②7⑧1⑩3⑩4⑪3
RJ	①1①4②1④3⑤1⑧2⑨1⑫1⑫2⑫5⑮2	GN	①3①6④1⑪2⑬4⑮6⑮8
HY	①2②9⑦2	GH	⑭2

^1^Circle numbers indicate a microsatellite locus with a character behind it (a unique allele type at that locus) composing a breed label.

^2^①: MCW0081; ②: MCW0213; ③: ADL0278; ④: MCW0330; ⑤: MCW0165; ⑥: MCW0020; ⑦: ADL0268; ⑧: MCW0016; ⑨: MCW0098; ⑩: MCW0123; ⑪: LEI0094; ⑫: LEI0166; ⑬: MCW0111; ⑭: MCW0104; ⑮: MCW0183.

On the Breed Label Sheet, there are many unique alleles of RJ in Chinese chicken breeds. The RJ chicken population has 11 unique alleles at eight loci, including MCW0081, MCW0213, MCW0330, MCW0165, MCW0016, MCW0098, LEI0166, and MCW0183. The unique allele frequency is much higher than that of other breeds. In addition to RJ, YL, and GN of Vietnamese breeds have seven unique alleles. Unlike RJ, YL showed unique alleles at two loci, LEI0094 and MCW0104, while GN showed unique alleles at two loci, LEI0094 and MCW0111. Therefore, LEI0094, MCW0104, and MCW0111 may be critical loci for distinguishing them.

## Discussion

Evaluation of the genetic diversity of species is mainly affected by the sampling process and sample size. Small sample sizes may lead to large errors in the genetic diversity of estimated species (Berthouly et al., 2009). Allele richness is one of the most important and commonly used estimators of genetic diversity in populations and is significantly affected by effective population size (Petit et al., 2008). Genetic analysis of 18 microsatellite loci revealed that the average number of alleles detected in 21 local flocks in Vietnam and China was 7.8, indicating that the experimental sample size was sufficient to reliably evaluate the genetic diversity of 21 local breeds (Pham et al., 2014). In addition, the average PIC content was 0.65, which showed moderate to high polymorphism (Vanhala et al., 1998), but also reflects large allelic diversity in the population. Observed heterozygosity (Obs He) in the whole population ranged from 0.4623 to 0.7193, which was similar to six previously reported local Indian chicken breeds (Pirany et al., 2007), the PIC values of CH, WX in Yunnan, and CT in Vietnam were relatively low, indicating that there were few genetic exchanges between these three local chicken breeds and other populations, possibly because CH was the closest breed to the red jungle fowl, while WX and CT had a narrower population genetic basis due to special morphological characteristics (Bao et al., 2008; Osei-Amponsah et al., 2010). The parameters of the other five local chicken breeds in Vietnam were higher than those of domestic local chicken breeds and maintained at a higher level, indicating that these five local chicken breeds in Vietnam may have experienced a better breeding process (Cuc et al., 2011). Overall, the chicken breeds in southern China and Vietnam represent unique genetic resources for modern poultry farming.

FIS indicates the degree of inbreeding among individuals within a population. In all microsatellite loci, MCW0111 and MCW0016 inbreeding coefficients were negative, indicating heterozygote excess, while other loci showed heterozygote deletion (Osei-Amponsah et al., 2010; Glazewska et al., 2018). FST among populations suggests that these chicken breeds are genetically highly differentiated (Acosta et al., 2013; Salamon et al., 2014). The mean value of the population fixation coefficient (FST) of the 21 local chicken populations was 0.167, indicating that 16.7% of the genetic variation in the population came from between populations, and other genetic variations were caused by individuals. These estimates also emerged from earlier studies, with a mean genetic differentiation (FST) of 0.085 for Vietnamese chicken breeds and 0.147 for Chinese chicken breeds. The value between the ­Chinese and Vietnamese breeds was 0.155 (Pham et al., 2013). Based on the allele frequencies of different samples at multiple loci, we used FST and Nei’s standard genetic distance method to measure the genetic differences between different subspecies which can directly reflect the genetic differentiation among varieties (Wright et al., 1968; Green et al., 1976). The difference in genetic distance caused by different levels of gene variation can be eliminated by standardizing the genetic distance, thus FST and DS are identical to a certain extent (Rousset, 1997; Meirmans, 2012). Our study showed that, except for CT Vietnamese chickens, the five breeds exhibited a lower genetic distance (*P* = 0.2425), and the corresponding FST was also lower (*P* = 0.0485), indicating that there may be a genetic exchange among them in Vietnam. The greatest genetic distance between varieties appeared between GH and WX, and correspondingly their pairwise FST also reached the highest value of 0.1834. The FST between most of the breeds was lower than 0.15. These results indicated that due to the geographical proximity, the genetic evolution of Chinese and Vietnamese chicken breeds was relatively slow, and genotype frequencies and kinship were highly correlated. In addition, this is difficult to distinguish because the genetic correlation between Chinese varieties is close and far. Therefore, we further reflected the genetic differentiation based on the neighbor-joining dendrogram of standard genetic distance (Nei et al., 1983). In the neighbor-joining dendrogram, the whole population was divided into four clusters, except for CH, CT, and WL, which were relatively far away from other populations, all populations were fine, indicating that the genetic diversity of the entire population was very abundant (Cuc et al., 2010). Among them, the genetic diversity of Guangxi chicken breeds was consistent with that of previous studies (Liao et al., 2016). The distance between GX and HY was relatively close, and the distance between XY and LS was relatively close. In addition, TC, YL, and WX in Yunnan and LS, XY in Guangxi were clustered together, respectively, which showed that there was a strong inbreeding relationship between chicken breeds in Yunnan and Guangxi, in addition to the consistent origin of ancestors, closer proximity leads to frequent communication between regions (Berthouly et al., 2009). CT in Vietnam also had a close genetic distance from the CH and WL chicken breeds in Yunnan, respectively, which indicates that there was genetic communication between Vietnamese and Yunnan chicken breeds in the early stage. This genetic communication is the result of many factors (Pham et al., 2013), the geographical location of domestication center regions (Liu et al., 2006), and the migration of humans and birds (Wang et al., 2021). Similarly, this idea was demonstrated by constructing a whole scatterplot using a PCoA analysis of 21 local chicken breeds.

In the present study, we used the structure program to analyze the population genetic structure of 21 local chicken breeds. It was found that Vietnamese local chicken breeds separated later from the population than some domestic local chicken breeds, indicating that some domestic local chicken breeds had a longer genetic differentiation time than five Vietnamese local chicken breeds, and also verified the history of ancient Chinese immigration to Vietnam (Savina, 1930). Previous studies showed that the best (∆*K*) for classifying the investigated breeds was *K* = 3 (Pritchard et al., 2000), while our results showed that at this time, the Vietnamese breeds CT had not separated from the Chinese chicken breeds, indicating that there was a strong consanguinity between the Vietnamese and Chinese chicken breeds due to the mixed breeds during long-term migration in southern China (Berthouly-Salazar et al., 2010), followed by the mixed breeds with wild and domestic chickens in the River Province (Berthouly et al., 2009). Vietnamese chickens showed high genetic diversity. In addition, until *K* = 12, some Chinese local chicken breeds were not separated, which indicates that some chicken breeds in Yunnan and Guangxi have high genetic diversity, which is consistent with the results of the neighbor-joining dendrogram Moreover, previous studies have shown that the heterozygosity of Yunnan local chicken breeds is higher than that of most populations in Africa, Europe, and Asia (Huo et al., 2014), which is similar to our results and may be the result of breeding methods used by people of different ethnic backgrounds living in complex environments and different economic conditions. Similar Vietnamese chicken breeds also showed a population separation of *K* = 5 under independent analysis, which suggests a high degree of mixing between Vietnamese chicken breeds. Previous studies have drawn similar conclusions (Cuc et al., 2010), while Vietnamese chicken breeds could be better separated at higher K values. The divergence of Vietnamese chicken breeds is closely related to their geographical distribution (Evelyne et al., 2022). Northern chicken breeds comprise an unstructured gene pool, and differentiation of Vietnamese chicken breeds can be observed between the northern and south-central coasts, as well as the Mekong Delta (Cuc et al., 2010).

Compared with other morphological and biochemical markers, microsatellite molecular markers can directly reflect changes at the DNA level, and their inheritance is not limited by the environment or gene expression (Lin and Chang, 2013). Currently, this is a commonly used method for identifying animal breeds and lines (Yang et al., 2021). In addition, other studies have found that microsatellites assigned chickens more correctly than any other marker type, 29 loci were better than 152 SNPs, however, using a higher number of SNPs would improve resolution (Granevitze et al., 2014). The cost of SNP analysis depends largely on the number of loci and individuals tested. Previous studies have shown 75 SNPs have the resolving power of 14 microsatellite loci, and SNP analysis with the same information content as microsatellite analysis is cost-effective only when a large number of individuals are tested simultaneously; otherwise, the development of molecular markers for random detection is expensive (Soglia et al., 2017).

Microsatellites have proven to be useful for identification in animals, and previous studies have shown that 98% kinship identification can be achieved by combining five polymorphic microsatellite loci (more than six alleles per locus), and 99.6% success can be achieved using ten such loci (Ellegren et al., 1992). Chicken breed-specific microsatellite markers have been constructed worldwide (Olschewsky and Hinrichs, 2021). For example, breed discrimination of local chicken breeds in Africa, Asia, and South America (Wimmers et al., 2000), as well as molecular markers of 20 breeds, including red original chickens, commercial chickens, and local chickens in Ukraine and Germany (Romanov and Weigend, 2001). In addition, the use of microsatellites to trace the source of meat has become an essential trend to protect the health of consumers (Dalvit et al., 2008). Previous studies used seven highly polymorphic microsatellite loci obtained to genotype six beef cattle in the Chinese market and found that the genotype coincidence probability of blood and ­corresponding tissues of each individual was 100% (Zhao et al., 2017). These studies indicated that the use of microsatellites plays an important role in the identification of breeds as well as individuals, and the discrimination power continues to increase with the gradual increase in the number of microsatellite markers (Herraeza et al., 2005) The effects of individual and breed visual errors were excluded using microsatellite analyses (Cunningham and Meghen, 2001). Our research on Vietnamese and Chinese local chicken breeds determined that they are geographically very close and may have some form of genetic relationship, therefore it is difficult to clearly distinguish them. We identified specific alleles at 15 microsatellite loci in 20 Vietnamese and Chinese landraces. However, due to the small number of microsatellite loci, only a small number of individuals in each breed have the unique alleles we identified. Therefore, we can only distinguish between populations through these unique alleles, but cannot ensure that all individuals in each population can be quickly distinguished. On this basis, follow-up research can further increase the frequency of microsatellite detection to achieve the level of rapid identification of individuals (Atul et al., 2014).

In conclusion, using microsatellite analysis, we found that the genetic diversity of chicken breeds in these 21 localities was high and had good breeding value, and there was a strong consanguinity mixture between some local southern chicken breeds in China. Vietnamese chickens are strongly associated with some local chicken breeds in Yunnan Province, China, and Vietnamese local chickens also have a high degree of hybridization, which is closely related to population movement and geographical connection. Finally, we constructed unique microsatellite recognition tags for breeds other than Vietnamese breed GM through unique alleles, providing an important basis for screening purebred chicken breeds for breed protection and subsequent breeding.

## Abbreviations:

CH: *Gallus gallus* spadiceus
CT: GÀ Tỉnh hưng
DS: Nei’s standard genetic distance
FAO: Food and Agriculture Organization of the United Nations
FIS: Within-subpopulation inbreeding coefficient
FIT: Total population inbreeding coefficient
FST: Among-subpopulation differentiation coefficient-gene fixation coefficient
GA: GÀ nòi LÔNG MÀU
GB: GÀ nòi LÔNG ĐEN
GH: Gà hồ
GM: GÀ MÍA
GN: GÀ TRÚNG XANH
GX: Guangxi partridge chicken
He: Expected heterozygosity
Ho: Observed heterozygosity
HY: Yao chicken
LP: Lanping silky chicken
LS: Longsheng feng chicken
Na: Observed number of alleles
Ne: Effective number of alleles
Nm: Gene flow
PCoA: Principal coordinate analysis
PIC: Olymorphism information content
PJ: Piao chicken
RJ: Red junglefowl, *Gallus gallus*
TC: Tengchong white chicken
UPGMA: Unweighted group average unweighted pair-group method with arithmetic mean
WD: Wuding chicken
WL: Wuliangshan black-bone chicken
WX: Daweishan mini chickens
XS: Xishuangbanna game chicken
XY: Xiayan chicken
YJ: Yanjin black-bone chicken
YL: Yunlong dwarf chicken
